# Albino *Xenopus laevis* tadpoles prefer dark environments compared to wild type

**DOI:** 10.17912/micropub.biology.000750

**Published:** 2023-02-06

**Authors:** Grace T Adebogun, Annabelle E Bachmann, Ashlyn A Callan, Ummara Khan, Amaris R Lewis, Alexa C Pollock, Sebastian A Alfonso, Daniel Arango Sumano, Dhruv A Bhatt, Aidan B Cullen, Cyrus M Hajian, Winnie Huang, Emma L Jaeger, Emily Li, A. Kaile Maske, Emma G Offenberg, Vy Ta, Waymon W Whiting, Jordan E McKinney, Julie Butler, Lauren A O’Connell

**Affiliations:** 1 BIO161 Organismal Biology Lab, Stanford University, Stanford, California, United States; 2 Department of Biology, Stanford University, Stanford, California, United States

## Abstract

Tadpoles display preferences for different environments but the sensory modalities that govern these choices are not well understood. Here, we examined light preferences and associated sensory mechanisms of albino and wild-type *Xenopus laevis* tadpoles. We found that albino tadpoles spent more time in darker environments compared to the wild type, although they showed no differences in overall activity. This preference persisted when the tadpoles had their optic nerve severed or pineal glands removed, suggesting these sensory systems alone are not necessary for phototaxis. These experiments were conducted by an undergraduate laboratory course, highlighting how *X. laevis* tadpole behavior assays in a classroom setting can reveal new insights into animal behavior.

**Figure 1. Albino Xenopus laevis tadpoles prefer darker environments compared to the wild type independent of eyes or pineal gland.**
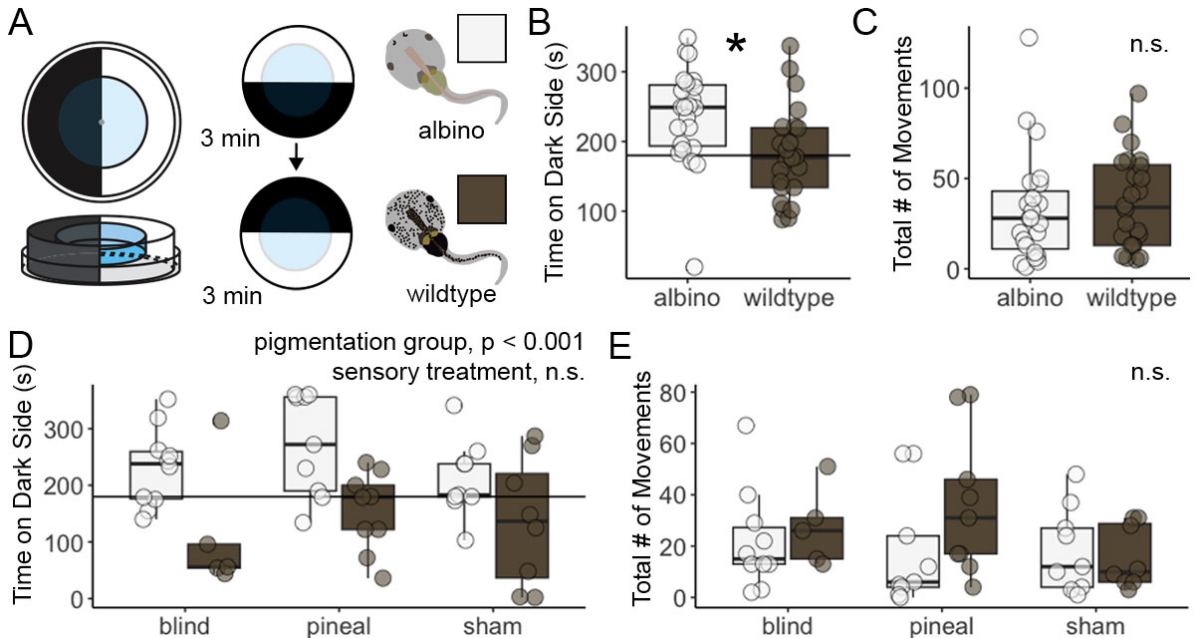
**(A)** Tadpole preferences for dark or light environments were tested in a simple chamber made of two petri dishes. The time spent on the dark side was recorded for three minutes before and after the outer chamber was rotated. **(B)** Albino tadpoles (white) spent more time in the darker environment compared to wild-type tadpoles (brown) (ANOVA, F(1) = 8.124, p = 0.007). **(C)** The total number of movements did not differ between groups. **(D)** Tadpole environmental preferences remained consistent despite being blind, having their pineal gland removed, or experiencing a control sham operation (2-way ANOVA, pigmentation group: F(1) = 16.448, p < 0.001; sensory treatment and their interaction was not significant). **(E)** The total number of movements did not differ between pigmentation groups nor sensory treatments.

## Description

Aquatic larvae display preferences for various visual environments, which may lead to increased survival and fitness by facilitating avoidance of predation and occupying habitats with more nutrients (Bai et al., 2016; Hettyey et al., 2012; Rot-Nikcevic et al., 2006). For example, tadpoles of the African clawed frog ( *Xenopus laevis* ) prefer green environments over dark or light environments (Hunt et al., 2020), which the authors suggest is an innate preference for plant-dense habitats that provide food and shelter. Amphibians have been used extensively over the last century to study visual systems (Donner and Yovanovich, 2020), and still remain an excellent research organism to link the development of sensory systems and neuronal circuits to adaptive behavioral outputs (Liu et al., 2016; Roberts et al., 2010). Here, we explore (1) using *X. laevis* phototaxis assays as a simple behavioral test for identifying light environment preferences and the sensory systems that mediate these responses and (2) using this approach in an undergraduate classroom to engage students in authentic scientific research. *Xenopus* tadpoles are easy to obtain and rear, have established genetic tools, and are amenable to simple behavioral tests like phototaxis assays that are robust and rapid. These features make it a promising organism for studying the sensory, neural, and physiological basis of environmental preferences within an undergraduate laboratory classroom.

Tadpoles make complex visually-guided decisions, including escaping from a looming stimulus (Dong et al., 2009) and light-mediated learning (Blackiston and Levin, 2013). Tadpole eyes and retinal projections to the brain begin to develop after hatching and mature following metamorphosis (Glücksmann, 1940). Visual acuity also increases across tadpole development, where avoidance of stimuli directed towards the eye correlates with the development of eye projections into the optic tectum (Dong et al., 2009). Despite this slow sensory maturation, tadpoles can respond to light throughout development. The pineal gland, a photoreceptive and endocrine region of the vertebrate brain (Mano and Fukada, 2007), can also sense light and induce changes in tadpole behavior (Foster and Roberts, 1982; Roberts, 1978). Moreover, most research in *X. laevis* utilizes either a pigmented wild-type line or an albino line that lacks pigmentation due to a mutation in the *hsp4* gene (HPS4, biogenesis of lysosomal organelles complex 3 subunit; Fukuzawa, 2021). Mutations of HSP4 in humans lead to oculocutaneous albinism (i.e., lack of pigment in the eyes, skin, and hair) in patients with Hermansky–Pudlak syndrome type 4 (OMIM:614073). Lack of melanin pigmentation can impact eye development and function, including reduced visual acuity and increased light sensitivity (Hertle, 2013). The impact of tadpole pigmentation differences on visual acuity and phototaxis behavior has not been studied in depth.

To examine the influence of pigmentation on light preferences, we tested the dark/light preferences of wild-type and albino *X. laevis* tadpoles using a chamber of concentric Petri dishes, in which the inner Petri dish holds the tadpole while the outer dish is painted black over half of its surface and can be rotated (Figure 1A). We found that albino tadpoles spent more time on the dark side compared to wild-type pigmented animals (Figure 1B; 1-way ANOVA, F(1) = 8.124, p = 0.007). These tadpole strains did not differ by total movement (Figure 1C; 1-way ANOVA, F(1) = 0.740, p = 0.394). Together, these data suggest that (1) albino tadpoles show a preference for darker environments, and (2) this pattern is not explained by differences in movement frequency between strains. Differences in visual sensitivities between albino and wild-type organisms have been reported in zebrafish (Ren et al., 2002), axolotls (Barr, 1988), and lab rodents (Balkema et al., 1981; Thomas et al., 2005), wherein albino animals demonstrate poorer vision in dim environments. Although albino tadpoles may not see well in dimmer conditions, darker environments may be preferred due to light sensitivity that is common in albinism (Hertle, 2013). Wild-type tadpoles did not display a preference for lighter or darker environments, which may be explained by the container type in which they were housed. A study by Moriya et al (1996) demonstrated that tadpoles reared in dark containers displayed a preference for dark environments, but no preference was found in tadpoles reared in white environments (Moriya et al., 1996). As the tadpoles in this study were housed in white containers, this may explain the lack of preference for light/dark environments in wild-type tadpoles. Future experiments could include the presentation of various colors or light conditions across development and with different housing conditions to further investigate environmental preferences across strains and how these may change as tadpole visual systems develop.

We next tested the hypothesis that either the eyes or the pineal gland are necessary for tadpoles to display a light/dark preference. Tadpoles were split into three groups that either had their optic nerves severed, their pineal gland removed, or underwent a control sham operation. After recovery, tadpoles were tested again in the phototaxis chamber. We found a significant effect of pigmentation group, but not sensory manipulation, on time spent on the dark side of the arena (Figure 1D; 2-way ANOVA, group: F(1) = 16.448, p < 0.001; treatment: F(2) = 1.148, p = 0.327; group*treatment: F(2) = 0.343, p = 0.712). Tadpoles did not differ in movement by pigmentation, sensory treatment, or their interaction (Figure 1D; 2-way ANOVA, group: F(1) = 2.892, p = 0.096; treatment: F(2) = 0.815, p = 0.449; group*treatment: F(2) = 1.214, p = 0.307). Together, these data suggest that neither eyes or the pineal gland alone are necessary for the albino tadpole preference for dark environments. An important caveat in this interpretation is that we did not confirm whether tadpoles were truly blind with additional behavior assays that are known to rely on retinal projections to the brain. However, other studies have indicated that eyes are not required for tadpole phototaxis responses (Butler et al., 2022) or responses to changes in light wavelength (Blackiston and Levin, 2013). Both of these studies suggest a role for the pineal gland in light detection, although our study suggests the pineal gland alone is not required for this behavior. It is possible that the eyes and pineal compensate for one another and a double ablation is necessary to influence phototaxis. Alternatively, skin could play a role in light detection, which was not investigated here. Amphibians possess photoreceptors on the skin (Becker and Cone, 1966), and *X. laevis* wild-type tadpoles will adjust the amount of pigmentation on their skin based on background coloration (Bertolesi et al., 2021).

In summary, we have shown that albino tadpoles prefer dark environments compared to the wild type and that eyes and the pineal gland alone are not required for phototaxis. Future studies should include double ablations of both eyes and the pineal gland as well as other sensory modalities to test alternative mechanisms of light environment preferences. Importantly, this work establishes the feasibility of using *X. laevis* phototaxis assays as a tool for learning more about animal behavior and sensory ecology within classroom undergraduate research experience courses.

## Methods

*Tadpoles*

Tadpoles were obtained from the National Xenopus Resource Center (see Reagents), including 27 wild type and 27 albino. Animals were maintained in a 1-gallon acrylic container with 0.1x Marc’s Modified Ringer's [MMR; 0.1 M NaCl, 2 mM KCl, 1 mM MgSO _4_ ,2 mM CaCl _2_ , 5 mM HEPES (pH 7.8), 0.1 mM EDTA] to a depth of 5 cm at 23C. Wild type and albino tadpoles were housed separately, and each container had an air stone and water recirculator. Tadpoles were fed a combination of crushed brine shrimp flakes and tadpole pellet food daily, with a 25% water change 4-6 hrs after feeding. All animals acclimated for 1 week prior to use in behavior experiments. Animals were stage 48-49 (Gerhart and Kirschner, 2020) in the first experiment. Four days after the initial test, tadpoles underwent surgeries to manipulate sensory systems relevant to phototaxis. Tadpoles were anesthetized in 0.01% MS-222 in buffered 0.1x MMR until loss of equilibrium and movement ceased. Blinded tadpoles had their optic nerves severed just caudal to the eye cup using a glass electrode. To remove the pineal organ, we used a NanoJect fitted with a glass electrode to gently dislodge and remove the pineal. The location of the pineal was identified by a circular break in pigmentation in wildtype tadpoles and based on location and visual identification in albino tadpoles. Sham tadpoles were anesthetized and poked with a glass electrode, similar to the blind and pinal-ablated tadpoles, but on the flank of the tadpole. All tadpoles were allowed to recover in 0.1x MMR for 24-48 hours. Tadpoles were stage 48-52 in the second experiment. All housing, surgical procedures, and behavior experiments were approved by the Stanford Administrative Panel on Laboratory Animal Care (#33097).

*Behavior Assays*

A behavioral arena was constructed using 10 cm and 15 cm Petri dishes. To create the light and dark environments, half of the 15 cm base was painted an opaque black, and a hole was drilled in the middle to allow a bolt to pass through. A 1 cm bolt was attached to the bottom of the 10 cm petri dish base, and a corresponding nut was attached to the inside of the 15 cm lid. To assemble, the 10cm base was screwed into the 15 cm lid, with the painted environment between the two. With this set-up, any necessary movement of the arena could occur using the 15 cm dish to ensure the tadpoles housed in the 10 cm dish could remain undisturbed. The arena was then placed on a light pad to illuminate the “light” side of the arena. To record the behavioral data, a recording device was placed above the arena using two boxes and a camera holder. The 10 cm dish was then filled with 0.1x MMR (~40 ml) and a label was placed near the arena to track tadpole identity.

Each tadpole was placed in the center of the arena for three minutes. The time tadpoles spent on the dark side of the arena and the total number of movements was recorded. After three minutes, the outer arena was rotated 180 degrees to flip the light and dark sides and behavior was recorded for another three minutes. After each session, the tadpoles were then weighed and measured, and their developmental stage was determined using Nieuwkoop and Fabor staging guide for *Xenopus* tadpoles (Gerhart and Kirschner, 2020). Tadpoles were placed back in their home canisters after the experiment was complete.

*Data Analysis*

Data analysis and visualization was performed in R (version 4.1.2). We first checked for outliers using Iglewicz and Hoaglin’s test for multiple outliers with a modified z-score of 3.5. Two outliers were detected in tadpole movement data of the sensory manipulation experiment and these individuals were excluded from the analyses. For the first experiment comparing wildtype and albino tadpoles, a one-way ANOVA was used to detect significant differences between groups, with either time spent on the dark side or the total movement as the dependent variable and tadpole group (wildtype or albino) as independent variables. For the second experiment with sensory manipulations, a two-way ANOVA was used to detect significant differences across pigmentation groups and sensory treatments, with either time spent on the dark side or the total movement as the dependent variable and tadpole group (wildtype or albino), treatment (blind, pineal gland removed, or sham operation) and their interaction as independent variables. Parametric assumptions were met by data on time spent on the dark side, including homogeneity of variance confirmed with Levene’s test (leveneTest) and normality of residuals confirmed with a Shapiro-Wilk test (shapiro.test). Total movement data did not originally meet the assumptions of a parametric test until the data was log-transformed. Boxplots were generated using the ggplot2 (version 3.3.5) package and assay illustrations were drawn in Adobe Illustrator (version 2023).

*Classroom pedagogy*

We conducted the experiments described here in two laboratory sessions. The first behavioral trial was preceded by a demonstration by the instructors. Eighteen students worked in pairs, where one student recorded time spent on the dark side of the arena with a time while the other student recorded the number of major movements with a tally counter. Weekly homework included reading relevant literature, data analysis and visualization, and writing the results and interpretations. The final project was to write this journal-style article.

## Reagents

**Table:** 

Strain Name	Genotype	Source
Wildtype *Xenopus laevis* tadpoles	Wild type	National Xenopus Resource at the Marine Biological Laboratory
Albino *Xenopus laevis* tadpoles	* Xla.NXT-WT:Albino ^NXR^*	National Xenopus Resource at the Marine Biological Laboratory
